# Finding an appropriate equation to measure similarity between binary vectors: case studies on Indonesian and Japanese herbal medicines

**DOI:** 10.1186/s12859-016-1392-z

**Published:** 2016-12-07

**Authors:** Sony Hartono Wijaya, Farit Mochamad Afendi, Irmanida Batubara, Latifah K. Darusman, Md Altaf-Ul-Amin, Shigehiko Kanaya

**Affiliations:** 1 Graduate School of Information Science, Nara Institute of Science and Technology, 8916-5 Takayama, Ikoma, Nara 630-0192 Japan; 2 Department of Computer Science, Bogor Agricultural University, Jl. Meranti Wing 20 Level 5 Kampus IPB Dramaga, Bogor, 16680 Indonesia; 3 Department of Statistics, Bogor Agricultural University, Jl. Meranti Wing 22 Level 4 Kampus IPB Dramaga, Bogor, 16680 Indonesia; 4 Tropical Biopharmaca Research Center, Bogor Agricultural University, Kampus IPB Taman Kencana, Jl. Taman Kencana No. 3, Bogor, 16128 Indonesia

**Keywords:** Binary data, Similarity measures, Distance metric, Jamu, Kampo, ROC curve, Hierarchical clustering

## Abstract

**Background:**

The binary similarity and dissimilarity measures have critical roles in the processing of data consisting of binary vectors in various fields including bioinformatics and chemometrics. These metrics express the similarity and dissimilarity values between two binary vectors in terms of the positive matches, absence mismatches or negative matches. To our knowledge, there is no published work presenting a systematic way of finding an appropriate equation to measure binary similarity that performs well for certain data type or application. A proper method to select a suitable binary similarity or dissimilarity measure is needed to obtain better classification results.

**Results:**

In this study, we proposed a novel approach to select binary similarity and dissimilarity measures. We collected 79 binary similarity and dissimilarity equations by extensive literature search and implemented those equations as an R package called bmeasures. We applied these metrics to quantify the similarity and dissimilarity between herbal medicine formulas belonging to the Indonesian Jamu and Japanese Kampo separately. We assessed the capability of binary equations to classify herbal medicine pairs into match and mismatch efficacies based on their similarity or dissimilarity coefficients using the Receiver Operating Characteristic (ROC) curve analysis. According to the area under the ROC curve results, we found Indonesian Jamu and Japanese Kampo datasets obtained different ranking of binary similarity and dissimilarity measures. Out of all the equations, the Forbes-2 similarity and the Variant of Correlation similarity measures are recommended for studying the relationship between Jamu formulas and Kampo formulas, respectively.

**Conclusions:**

The selection of binary similarity and dissimilarity measures for multivariate analysis is data dependent. The proposed method can be used to find the most suitable binary similarity and dissimilarity equation wisely for a particular data. Our finding suggests that all four types of matching quantities in the Operational Taxonomic Unit (OTU) table are important to calculate the similarity and dissimilarity coefficients between herbal medicine formulas. Also, the binary similarity and dissimilarity measures that include the negative match quantity *d* achieve better capability to separate herbal medicine pairs compared to equations that exclude *d*.

**Electronic supplementary material:**

The online version of this article (doi:10.1186/s12859-016-1392-z) contains supplementary material, which is available to authorized users.

## Background

Binary features have been commonly used to represent a great variety of data [1–3], expressing the binary status of samples as presence/absence, yes/no, or true/false. It has many applications in the bioinformatics, chemometrics, and medical fields [4–19], as well as in pattern recognition, information retrieval, statistical analysis, and data mining [20, 21]. The choice of an appropriate coefficient of similarity or dissimilarity is necessary to evaluate multivariate data represented by binary feature vectors because different similarity measures may yield conflicting results [22]. Choi et al. [23] collected binary similarity and dissimilarity measures used over the last century and revealed their correlation through the hierarchical clustering technique. They also classified equations into two groups based on inclusion and exclusion of negative matches. Consonni & Todeschini [1] proposed five new similarity coefficients and compared those coefficients with some well-known similarity coefficients. Three of the five similarity coefficients are less correlated with the other common similarity coefficients and need an investigation to understand their potential. Meanwhile, Todeschini et al. [24] reported an analysis of 44 different similarity coefficients for computing the similarities between binary fingerprints by using simple descriptive statistics, correlation analysis, multidimensional scaling Hasse diagrams, and their proposed method ‘atemporal target diffusion model’.

Nowadays, the utilization of herbal medicines, i.e. Indonesian Jamu, Japanese Kampo, traditional Chinese medicine (TCM), and so on [25], are becoming popular for disease treatment and maintaining good health. In case of Indonesian Jamu, each Jamu medicine is prepared from a single plant or a mixture of several plants as its ingredients. The National Agency of Drug and Food Control (NA-DFC) of Indonesia supervises the production of Jamu medicines before its release for public use. Up to 2014, there were 1247 Jamu factories in Indonesia [26]. They have concocted a lot of Jamu formulas with various efficacies. Consequently, the studies of Jamu formulas have become an interesting research topic in the last few years. It may be related to the problems of the Jamu philosophy, systematization of Jamu, or phytochemistry. In the Jamu studies, the relationships between plants, Jamu, and efficacies lead to determine important plants for every disease class using global and local approaches [4, 5, 27]. In addition, Kampo formulas are traditional medicines from Japan. These are generally prepared by combination of crude drugs. In total, 294 Kampo formulas are listed in the Japanese Pharmacopoeia of 2012 and it can be used for self-medication [28]. Currently, many researchers have done Kampo studies to unveil the complex systems of Kampo medication and to reveal the scientific aspect of its relevance to modern healthcare. In Jamu and Kampo studies, herbal medicine formula and plant/crude drug relations are represented as binary feature vectors, denoting whether a particular plant is used or not as an ingredient.

The relationships between Jamu formulas, as well as Kampo formulas and other herbal medicines, are not only reflected by the efficacy similarity but also by the ingredient similarity. One Jamu formula can be suggested as an alternative to the other one if they have relatively similar ingredients. For mathematical analysis, each Jamu formula is represented as a binary vector using 1 to indicate the presence of a plant and 0 otherwise. However, each Jamu formula usually uses a few plants. Thus, most of the Jamu vectors contain a few 1 s and many 0 s. Consequently, the number of plants that are used simultaneously in Jamu pairs is much smaller than the number of plants that are not used simultaneously as Jamu ingredients. Therefore, in order to find relatively similar Jamu formulas, the high number of negative matches might influence the calculation of binary similarity or dissimilarity between Jamu pairs. On the other hand, there is no guarantee that negative co-occurrence between two entities is identical [29]. Hence, it is necessary to examine the binary similarity and dissimilarity coefficients of Jamu formulas to determine the appropriate measurement for finding a suitable mixing alternative of a target crude drug.

Currently, there are several methods to measure the quality of classifiers [30, 31] such as the Receiver Operating Characteristic (ROC) curves [32, 33], Precision-Recall (PR) curves [33, 34], Cohen’s Kappa scores [35, 36], and so on. An ROC curve is a very powerful tool for measuring classifiers’ performance in many fields, especially in the machine learning and binary-class problems [37]. The purpose of ROC analysis is similar to that of the Cohen’s Kappa, which is mainly used for ranking classifiers. The ROC curve conveys more information than Cohen’s Kappa in a sense that it can also visualize the performance of a classifier by a curve instead of generating just a scalar value. In this study, we propose a method to select the most suitable similarity measures in the context of classification based on False Positive Rates (FPRs) and True Positive Rates (TPRs) by using ROC curve analysis. We discuss the step-by-step development of this method by applying it to assess the similarity of herbal medicines in the context of their efficacies. Initially, we gathered 79 binary similarity and dissimilarity equations. Some identical equations were eliminated in the preliminary step. Subsequently, the capability of binary measures to separate herbal medicine pairs into match and mismatch efficacy groups was assessed by using the ROC analysis.

## Methods

**Fig. 1:**
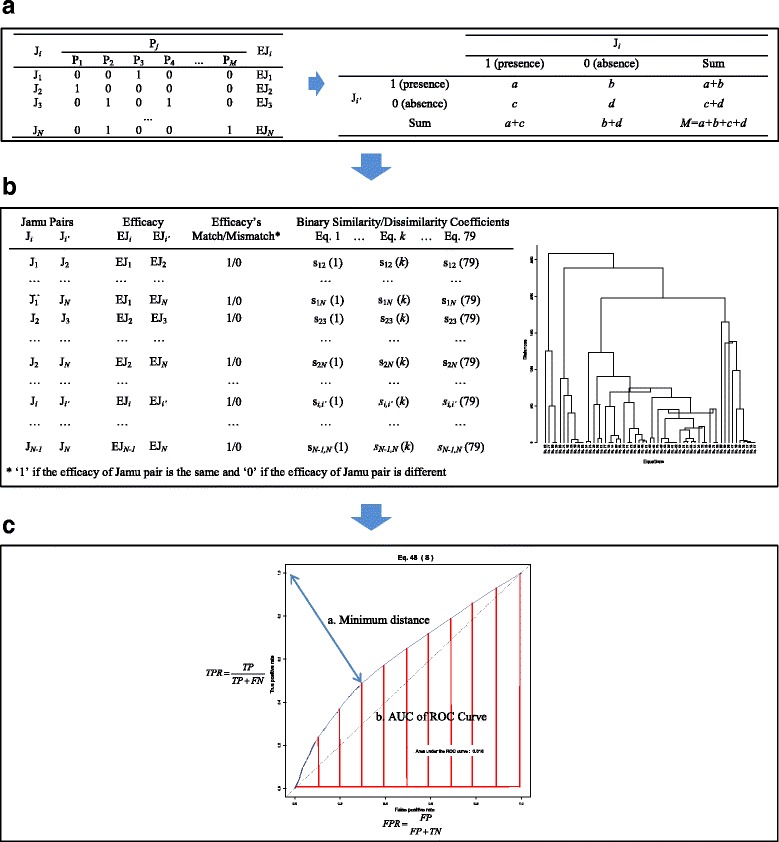
An illustration of the experimental flow. This figure also illustrates representation of plant, herbal medicine formulas and efficacy relations as two-dimensional matrix. **a** Format of the input data representing Jamu-plant relations and the OTUs expression of a Jamu pair. **b** Reducing the candidate equations. **c** The ROC analysis

The proposed method leads to the selection of a suitable equation such that when two herbal medicine formulas belong to the same efficacy group, their ingredient similarity measured by the equation becomes higher in the global context of a large set of formulas. Figure 1 illustrates data representation and also the procedure of our experiment.

### Datasets

We used 3131 Jamu formulas collected from NA-DFC of Indonesia [4, 5, 27], which comprise of 465 plants. Thus, Jamu vs. plant relations were then organized as a 3131x465 matrix (Fig. 1a). Jamu formulas were represented by binary vectors, which express the binary status of plants as ingredients, 1 (presence) and 0 (absence). Each Jamu formula consists of 1 to 26 plants, with average 4.904, standard deviation 2.969 and the set union of all formulas consists of 465 plants. Each Jamu formula corresponds to one or more efficacy/disease classes. Total 14 disease classes are used in this Jamu study, of which 12 classes are from the National Center for Biotechnology Information (NCBI) [38]. The list of disease classes are as follows: blood and lymph diseases (E1), cancers (E2), the digestive system (E3), female-specific diseases (E4), the heart and blood vessels (E5), diseases of the immune system (E6), male-specific diseases (E7), muscle and bone (E8), the nervous system (E9), nutritional and metabolic diseases (E10), respiratory diseases (E11), skin and connective tissue (E12), the urinary system (E13), and mental and behavioral disorders (E14). Corresponding to 3131 Jamu formulas, there can be (3,131x3,130)/2 = 4,900,015 Jamu pairs.

For the purpose of comparison, we created four random matrices as the same size as Jamu-plant relations by randomly inserting 1 s and 0 s. In three of the random datasets, the numbers of 1 s are 1, 5 and 10% of 465 plants (called as random 1%, random 5%, and random 10%). In the case of the other dataset, we randomly inserted the equal number of 1 s in every row as it is in the original Jamu formulas (called as random Jamu). We also applied our proposed method into Kampo dataset [28]. This dataset is presented as a two-dimensional binary matrix with rows and columns representing Kampo formulas and crude drug ingredients, respectively. Kampo dataset is composed of 274 Kampo formulas and each formula consists of 3 to 19 crude drugs, with average 8.923, standard deviation 3.885, and the set union of all formulas consists of 227 crude drugs. Then, each Kampo formula is classified into deficiency or excess class, according to Kampo-specific diagnosis of patient’s constitution.

### Flow of the experiment

The binary similarity (S) and dissimilarity (D) measure between a herbal medicine pair is expressed by the Operational Taxonomic Units (OTUs as shown in Fig. 1a) [39, 40]. Concretely, let two Jamu formulas be described by two-row vectors *J*_*i*_ and *J*_*i’*_, each comprised of *M* variables with value 1 (presence) or 0 (absence). The four quantities *a, b, c, d* in the OTUs table are defined as follows: *a* is the number of features where the values for both *j*_*i*_ and *j*_*i’*_ are 1 (positive matches), *b* and *c* are the number of features where the value for *j*_*i*_ is 0 and *j*_*i’*_ is 1 and vice versa, respectively (absence mismatches), and *d* is the number of features where the values for both *j*_*i*_ and *j*_*i’*_ are 0 (negative matches). The sum of *a* and *d* represents the total number of matches between *j*_*i*_ and *j*_*i’*_, the sum of *b* and *c* represents the total number of mismatches between *j*_*i*_ and *j*_*i’*_. The total sum of the quantities in the OTUs table *a* + *b* + *c* + *d* is equal to *M*.

We collected equations to measure similarity or dissimilarity between binary vectors from literature [1, 3, 20, 21, 23, 24, 29, 40–62], listed as Eqs. 1-79 in Table 1. The binary similarity and dissimilarity equations were represented by four quantities, i.e. *a, b, c* and *d*. We also implemented these 79 equations as an R package, called bmeasures. The bmeasures package is available on Github and can be installed by invoking these commands: install.packages(“devtools”), library(“devtools”), install_github(“shwijaya/bmeasures”), library(“bmeasures”). The installation of bmeasures package was tested on R release 3.2.4 and the devtools package ver. 1.11.0. Initially, we measure the similarity and dissimilarity coefficients between herbal medicine pairs by using 79 equations. Then, the resulted similarity/dissimilarity coefficients are used for further analysis. Our experimental procedure can be divided into two major steps, which we discuss in the following segments:

**Table 1:** List of 79 binary similarity and dissimilarity measures

Eq. IDs	Equations	References	Note
1	${S}_{Jaccard}=\frac{a}{a+b+c}$	[1, 20, 21, 23, 24, 29, 40–43, 45–50, 55]	
2	${S}_{Dice-2}=\frac{a}{2a+b+c}$	[20, 21, 47, 48]	
3	${S}_{Dice-1/ Czekanowski}=\frac{2a}{2a+b+c}$	[3, 23, 24, 29, 40–42, 44–47, 49, 50, 55]	***
4	${S}_{3W- Jaccard}=\frac{3a}{3a+b+c}$	[23, 24, 43, 47]	
5	${S}_{Nei\&Li}=\frac{2a}{\left(a+b\right)+\left(a+c\right)}$	[23, 40, 54]	*
6	${S}_{Sokal\& Sneath-1}=\frac{a}{a+2b+2c}$	[1, 23, 24, 40, 45, 47, 55]	
7	${S}_{Sokal\& Michener}=\frac{a+d}{a+b+c+d}$	[1, 3, 20, 21, 23, 24, 29, 40–42, 45, 46, 48–50]	
8	${S}_{Sokal\& Sneath-2}=\frac{2\left(a+d\right)}{2a+b+c+2d}$	[1, 23, 24, 40, 45, 49, 50, 55]	
9	${S}_{Roger\& Tanimoto}=\frac{a+d}{a+2\left(b+c\right)+d}$	[20, 21, 23, 24, 29, 40, 41, 45, 46, 48–50, 55, 56]	
10	${S}_{Faith}=\frac{a+0.5d}{a+b+c+d}$	[23, 24, 56, 57]	
11	${S}_{Gower\& Legendre}=\frac{a+d}{a+0.5\left(b+c\right)+d}$	[23, 24, 58]	*
12	*S*_*Intersection*_ = *a*	[23, 47]	
13	*S*_*Innerproduct*_ = *a* + *d*	[23]	***
14	${S}_{Russell\&Rao}=\frac{a}{a+b+c+d}$	[1, 3, 20, 21, 23, 24, 29, 40, 41, 45, 47–50, 55, 56]	***
15	*D*_*Hamming*_ = *b* + *c*	[23, 48, 59]	
16	${D}_{Euclid}=\sqrt{b+c}$	[23]	
17	${D}_{Squared- euclid}=\sqrt{{\left(b+c\right)}^2}$	[23, 60]	*
18	${D}_{Canberra}={\left(b+c\right)}^{\frac{2}{2}}$	[23]	*
19	*D*_*Manhattan*_ = *b* + *c*	[23]	*
20	${D}_{Mean- Manhattan}=\frac{b+c}{a+b+c+d}$	[23, 55]	***
21	*D*_*Cityblock*_ = *b* + *c*	[23]	*
22	${D}_{Minkowski}={\left(b+c\right)}^{\frac{1}{1}}$	[23]	*
23	${D}_{Vari}=\frac{b+c}{4\left(a+b+c+d\right)}$	[23, 61]	***
24	${D}_{SizeDifference}=\frac{{\left(b+c\right)}^2}{{\left(a+b+c+d\right)}^2}$	[23]	
25	${D}_{ShapeDifference}=\frac{n\left(b+c\right)-{\left(b-c\right)}^2}{{\left(a+b+c+d\right)}^2}$	[23]	
26	${D}_{PatternDifference}=\frac{4bc}{{\left(a+b+c+d\right)}^2}$	[23]	
27	${D}_{Lance\& Williams}=\frac{b+c}{2a+b+c}$	[23, 61]	
28	${D}_{Bray\& Curtis}=\frac{b+c}{2a+b+c}$	[23]	*
29	${D}_{Hellinger}=2\sqrt{\left(1-\frac{a}{\sqrt{\left(a+b\right)\left(a+c\right)}}\right)}$	[23]	
30	${D}_{Chord}=\sqrt{2\left(1-\frac{a}{\sqrt{\left(a+b\right)\left(a+c\right)}}\right)}$	[23]	***
31	${S}_{Cosine}=\frac{a}{\sqrt{\left(a+b\right)\left(a+c\right)}}$	[24, 55]	
32	${S}_{Gilbert\& Wells}= \log a- \log n- \log \left(\frac{a+b}{n}\right)- \log \left(\frac{a+c}{n}\right)$	[23, 45]	**
33	${S}_{Ochiai-1}=\frac{a}{\sqrt{\left(a+b\right)\left(a+c\right)}}$	[23, 24, 29, 40, 41, 49, 55, 56]	*
34	${S}_{Forbes-1}=\frac{na}{\left(a+b\right)\left(a+c\right)}$	[23, 24, 40, 45, 47, 55]	
35	${S}_{Fossum}=\frac{n{\left(a-0.5\right)}^2}{\left(a+b\right)\left(a+c\right)}$	[23, 24, 55]	
36	${S}_{Sorgenfrei}=\frac{a^2}{\left(a+b\right)\left(a+c\right)}$	[23, 24, 40, 45]	
37	${S}_{Mountford}=\frac{a}{0.5\left( ab+ac\right)+bc}$	[23, 24, 40, 45]	**
38	${S}_{Otsuka}=\frac{a}{{\left(\left(a+b\right)\left(a+c\right)\right)}^{0.5}}$	[23, 46]	*
39	${S}_{McConnaughey}=\frac{a^2-bc}{\left(a+b\right)\left(a+c\right)}$	[23, 40, 45, 55]	
40	${S}_{Tarwid}=\frac{na-\left(a+b\right)\left(a+c\right)}{na+\left(a+b\right)\left(a+c\right)}$	[23, 45]	
41	${S}_{Kulczynski-2}=\frac{\frac{a}{2}\left(2a+b+c\right)}{\left(a+b\right)\left(a+c\right)}$	[23, 40, 45, 46, 49, 55]	***
42	${S}_{Driver\& Kroeber}=\frac{a}{2}\left(\frac{1}{a+b}+\frac{1}{a+c}\right)$	[23, 40, 45]	***
43	${S}_{Johnson}=\frac{a}{a+b}+\frac{a}{a+c}$	[23, 24, 40, 45, 51]	***
44	${S}_{Dennis}=\frac{ad-bc}{\sqrt{n\left(a+b\right)\left(a+c\right)}}$	[23, 24, 55]	
45	${S}_{Simpson}=\frac{a}{ \min \left(a+b,a+c\right)}$	[23, 24, 40, 45, 55]	
46	${S}_{Braun\& Banquet}=\frac{a}{ \max \left(a+b,a+c\right)}$	[23, 24, 40, 45, 47]	
47	${S}_{Fager\& McGowan}=\frac{a}{\sqrt{\left(a+b\right)\left(a+c\right)}}-\frac{ \max \left(a+b,a+c\right)}{2}$	[23, 45]	
48	${S}_{Forbes-2}=\frac{na-\left(a+b\right)\left(a+c\right)}{n \min \left(a+b,a+c\right)-\left(a+b\right)\left(a+c\right)}$	[23, 45]	
49	${S}_{Sokal\& Sneath-4}=\frac{\frac{a}{\left(a+b\right)}+\frac{a}{\left(a+c\right)}+\frac{d}{\left(b+d\right)}+\frac{d}{\left(c+d\right)}}{4}$	[1, 24, 40, 45]	
50	${S}_{Gower}=\frac{a+d}{\sqrt{\left(a+b\right)\left(a+c\right)\left(b+d\right)\left(c+d\right)}}$	[23]	
51	${S}_{Pearson-1}={\chi}^2=\frac{n{\left( ad-bc\right)}^2}{\left(a+b\right)\left(a+c\right)\left(c+d\right)\left(b+d\right)}$	[23, 40, 45]	
52	${S}_{Pearson-2}={\left(\frac{\chi^2}{n+{\chi}^2}\right)}^{\frac{1}{2}}$	[23, 45]	
53	${S}_{Pearson-3}={\left(\frac{\rho }{n+\rho}\right)}^{\frac{1}{2}}$ $\mathrm{where}\kern0.75em \rho =\frac{ad-bc}{\sqrt{\left(a+b\right)\left(a+c\right)\left(b+d\right)\left(c+d\right)}}$	[23]	**
54	${S}_{Pearson\& Heron-1}=\frac{ad-bc}{\sqrt{\left(a+b\right)\left(a+c\right)\left(b+d\right)\left(c+d\right)}}$	[20, 21, 23, 24, 40, 45]	
55	${S}_{Pearson\& Heron-2}= \cos \left(\frac{\pi \sqrt{bc}}{\sqrt{ad}+\sqrt{bc}}\right)$	[23, 45]	
56	${S}_{Sokal\& Sneath-3}=\frac{a+d}{b+c}$	[23, 40, 45, 55]	**
57	${S}_{Sokal\& Sneath-5}=\frac{ad}{\left(a+b\right)\left(a+c\right)\left(b+d\right){\left(c+d\right)}^{0.5}}$	[1, 23, 24, 40, 45]	
58	${S}_{Cole}=\frac{\sqrt{2}\left( ad-bc\right)}{\sqrt{{\left( ad-bc\right)}^2-\left(a+b\right)\left(a+c\right)\left(b+d\right)\left(c+d\right)}}$	[23, 45]	**
59	${S}_{Stiles}={ \log}_{10}\frac{n{\left(\left| ad-bc\right|-\frac{n}{2}\right)}^2}{\left(a+b\right)\left(a+c\right)\left(b+d\right)\left(c+d\right)}$	[23, 40, 53, 55]	
60	${S}_{Ochiai-2}=\frac{ad}{\sqrt{\left(a+b\right)\left(a+c\right)\left(b+d\right)\left(c+d\right)}}$	[23, 29, 49]	*
61	${S}_{Yuleq}=\frac{ad-bc}{ad+bc}$	[20, 21, 23, 24, 40, 41, 45, 46, 48, 55]	
62	${D}_{Yuleq}=\frac{2bc}{ad+bc}$	[23]	
63	${S}_{Yulew}=\frac{\sqrt{ad}-\sqrt{bc}}{\sqrt{ad}+\sqrt{bc}}$	[3, 23, 24, 40, 45]	
64	${S}_{Kulczynski-1}=\frac{a}{b+c}$	[3, 20, 21, 23, 45–50, 55]	**
65	${S}_{Tanimoto}=\frac{a}{\left(a+b\right)+\left(a+c\right)-a}$	[1, 23, 24, 55]	*
66	${S}_{Disperson}=\frac{ad-bc}{{\left(a+b+c+d\right)}^2}$	[23, 24]	
67	${S}_{Hamann}=\frac{\left(a+d\right)-\left(b+c\right)}{a+b+c+d}$	[3, 23, 40, 45, 46, 49, 50, 55]	***
68	${S}_{Michael}=\frac{4\left( ad-bc\right)}{{\left(a+d\right)}^2+{\left(b+c\right)}^2}$	[23, 24, 40, 45, 52]	
69	${S}_{Goodman\& Kruskal}=\frac{\sigma -{\sigma}^{\hbox{'}}}{2n-{\sigma}^{\hbox{'}}}$ $$ \begin{array}{l}\mathrm{where}\;\sigma = \max \left(a,b\right)+ \max \left(c,d\right)+ \max \left(a,c\right)+ \max \left(b,d\right)\\ {}\kern1.56em {\sigma}^{\hbox{'}}= \max \left(a+c,b+d\right)+ \max \left(a+b,c+d\right)\end{array} $$	[23]	**
70	${S}_{Anderberg}=\frac{\sigma -{\sigma}^{\hbox{'}}}{2n}$	[23]	**
71	${S}_{Baroni- Urbani\& Buser-1}=\frac{\sqrt{ad}+a}{\sqrt{ad}+a+b+c}$	[23, 24, 40, 45, 55, 56, 62]	
72	${S}_{Baroni- Urbani\& Buser-2}=\frac{\sqrt{ad}+a-\left(b+c\right)}{\sqrt{ad}+a+b+c}$	[23, 24, 40, 45, 62]	***
73	${S}_{Peirce}=\frac{ab+bc}{ab+2bc+ cd}$	[23, 45]	**
74	${S}_{Eyraud}=\frac{n^2\left(na-\left(a+b\right)\left(a+c\right)\right)}{\left(a+b\right)\left(a+c\right)\left(b+d\right)\left(c+d\right)}$	[23]	
75	${S}_{Tarantula}=\frac{\frac{a}{\left(a+b\right)}}{\frac{c}{\left(c+d\right)}}=\frac{a\left(c+d\right)}{c\left(a+b\right)}$.	[23]	**
76	${S}_{Ample}=\left|\frac{\frac{a}{\left(a+b\right)}}{\frac{c}{\left(c+d\right)}}\right|=\left|\frac{a\left(c+d\right)}{c\left(a+b\right)}\right|$.	[23]	**
77	${S}_{Derived\_ Rusell-Rao}=\frac{ \log \left(1+a\right)}{ \log \left(1+n\right)}$.	[1, 24]	
78	${S}_{Derived\_ Jaccard}=\frac{ \log \left(1+a\right)}{ \log \left(1+a+b+c\right)}$	[1, 24]	
79	${S}_{Var\_ of\_ Correlation}=\frac{ \log \left(1+ ad\right)- \log \left(1+bc\right)}{ \log \left(1+{n}^2/4\right)}$	[1, 24]	

*S* is similarity measure, *D* is dissimilarity measure, *means algebraically redundant, **means produce infinite/NaN coefficients or indeterminate forms, ***means grouped in the same cluster with zero or nearly to zero distance, *n* is a constant (*n* = *M* = *a* + *b* + *c* + *d*)

**Step 1.** Reducing the candidate equations

The binary similarity and dissimilarity equations were evaluated to eliminate duplications. When two or more equations can be transformed into the same form by algebraic manipulations, only one of them is kept for further analysis. We also removed equations from our analysis that produce infinite/NaN values or indeterminate forms while applying to measure similarity and dissimilarity using all datasets.

Hierarchical clustering of the remaining equations was then done with an aim to further narrow down the number of candidate equations and to evaluate the closeness between equations. After we obtained the similarity/dissimilarity coefficients between herbal medicine pairs for each equation, we clustered those equations based on its similarity/dissimilarity coefficients using Agglomerative hierarchical clustering with Centroid linkage (Fig. 1b) [50, 63–65]. The Euclidean distance (Eq. 80) was used to measure the distance between two equations, *k* and *l*, that is:

80 $$ {d}_{k,l}=\sqrt{{\displaystyle {\sum}_{m=1}^{N-1}}{\displaystyle {\sum}_{n=m+1}^N}{\left({s}_{mn}(k)-{s}_{mn}(l)\right)}^2} $$

where *s*_*mn*_(*k*) and *s*_*mn*_(*l*) are the similarity/dissimilarity values between corresponding herbal medicine pair using equations *k* and *l* respectively, *N* is the total number of herbal medicine formulas, and *d*_*k*,*l*_ is the distance between equation *k* and *l*. The cluster centroid is the average values of the variables for the observations (in the present case equations) in that cluster. Let ${\overline{X}}_G,{\overline{X}}_H$ denote group averages for clusters *G* and *H*. Then, the distance between cluster centroids is calculated using Eq. 81.

81 $$ {d}_{centroid}\left(G,H\right)=\left\Vert {\overline{X}}_G\right.-{\left.{\overline{X}}_H\right\Vert}_2 $$

where ${\overline{X}}_G$ is the centroid of *G* by arithmetic mean ${\overline{X}}_G=\frac{1}{n_G}{\displaystyle {\sum}_{i=1}^{n_G}}{X}_{Gi}$ [2, 65, 66]. We implemented the clustering process using hclust function in R. At each step, the cluster centroid was calculated to represent a group of equations in the clusters. Furthermore, two equations or clusters are merged for which the distance between the centroids is the minimum until all equations are merged into one cluster.

We performed the hierarchical clustering process twice, first to reduce the candidate equations for which the distance between equations measured by Eq. 80 is zero or nearly zero and secondly to evaluate the combined characteristic of a group of equations. Mean centering and unit variance scaling was applied to the similarity/dissimilarity coefficients before the clustering process.

**Step 2.** ROC Analysis of selected equations

The effectiveness of similarity/dissimilarity measuring capability of the selected equations was evaluated by means of the ROC curve (Fig. 1c) [67, 68]. For ROC analysis, we divided all the herbal medicine pairs into match and mismatch efficacy classes and used the corresponding distributions with respect to similarity scores to calculate FPRs and TPRs. The ROC curve was created by selecting a series of threshold to generate *FPR* and *TPR. FPR* is the proportion of false positive predictions out of all the false data and *TPR* is the proportion of true positive predictions out of all the true data, defined by Eq. 82 [67–69]:

82 $$ FPR=FP/\left(FP+TN\right)\kern2em TPR=TP/\left(TP+FN\right) $$

where true positive (*TP*) is the number of herbal medicine pairs correctly classified as positive, true negative (*TN*) is the number of pairs correctly classified as negative, false positive (*FP*) is the number of pairs incorrectly classified as positive, and false negative (*FN*) is the number of pairs incorrectly classified as negative. We defined and compared the performance of good equations by using the minimum distance of the ROC curve to the theoretical optimum point and by using the Area Under the ROC Curve (AUC) analysis [70]. The minimum distance between the ROC curve and the optimum point was measured as the Euclidean distance. The minimum distance can also be computed by *TP*, *TN*, *FP*, and *FN* values corresponding to selected similarity thresholds *i* using the following formulation:

83 $$ Min.\  dist={ \min}_{i\ \in\ thresholds}\sqrt{{\left(F{P}_i/\left(T{N}_i+F{P}_i\right)\right)}^2+{\left(F{N}_i/\left(T{P}_i+F{N}_i\right)\right)}^2} $$

## Results and discussion

### Preliminary verification of the equations

In the preliminary step, we removed 12 equations denoted by ‘*’ in Table 1 because each of them can be recognized as identical to one or more other equations by only algebraic manipulations such as linear transformation. From the seven groups of redundant equations shown in Table 2, we included S_Jaccard_, S_Dice-1/Czekanowski_, S_Sokal_&_Sneath-2_, D_Hamming_, D_Lance_&_Williams_, S_Cosine_ and S_Sokal_&_Sneath-5_ in our analysis and therefore, we were left with 67 equations at this stage. Next, we clustered the 67 equations to reduce the number of equations using Jamu and Kampo datasets. During the clustering process, we eliminated 11 equations indicated by ‘**’ in Table 1 that produced infinite/NaN values or indeterminate forms while applied to all datasets. Such conditions can be reached when denominator of an equation becomes equal to 0, i.e. the values of *b* and *c* in the Mountford and Peirce similarities (Eq. 37 and Eq. 73) are 0 if two formulas use exactly the same ingredients.

**Table 2:** Groups of identical equations

Groups	Eliminated Equations	Selected Equations
1	${S}_{Nei\&Li}=\frac{2a}{\left(a+b\right)+\left(a+c\right)}$ (Eq.5)	${S}_{Dice-1/ Czekanowski}=\frac{2a}{2a+b+c}$ (Eq.3)
2	${S}_{Gower\& Legendre}=\frac{a+d}{a+0.5\left(b+c\right)+d}$ (Eq.11)	${S}_{Sokal\& Sneath-2}=\frac{2\left(a+d\right)}{2a+b+c+2d}$ (Eq.8)
3	${D}_{Squared- euclid}=\sqrt{{\left(b+c\right)}^2}$ (Eq.17)	*D*_*Hamming*_ = *b* + *c* (Eq.15)
	$$ {D}_{Canberra}={\left(b+c\right)}^{\frac{2}{2}} $$ (Eq.18)	
	*D*_*Manhattan*_ = *b* + *c* (Eq.19)	
	*D*_*Cityblock*_ = *b* + *c* (Eq.21)	
	$$ {D}_{Minkowski}={\left(b+c\right)}^{\frac{1}{1}} $$ (Eq.22)	
4	${D}_{Bray\& Curtis}=\frac{b+c}{2a+b+c}$ (Eq.28)	${D}_{Lance\& Williams}=\frac{b+c}{2a+b+c}$ (Eq.27)
5	${S}_{Ochiai-1}=\frac{a}{\sqrt{\left(a+b\right)\left(a+c\right)}}$ (Eq.33)	${S}_{Cosine}=\frac{a}{\sqrt{\left(a+b\right)\left(a+c\right)}}$ (Eq.31)
	$$ {S}_{Otsuka}=\frac{a}{{\left(\left(a+b\right)\left(a+c\right)\right)}^{0.5}} $$ (Eq.38)	
6	${S}_{Ochiai-2}=\frac{ad}{\sqrt{\left(a+b\right)\left(a+c\right)\left(b+d\right)\left(c+d\right)}}$ (Eq.60)	${S}_{Sokal\& Sneath-5}=\frac{ad}{\left(a+b\right)\left(a+c\right)\left(b+d\right){\left(c+d\right)}^{0.5}}$ (Eq.57)
7	${S}_{Tanimoto}=\frac{a}{\left(a+b\right)+\left(a+c\right)-a}$ (Eq.65)	${S}_{Jaccard}=\frac{a}{a+b+c}$ (Eq.1)

**Fig. 2:**
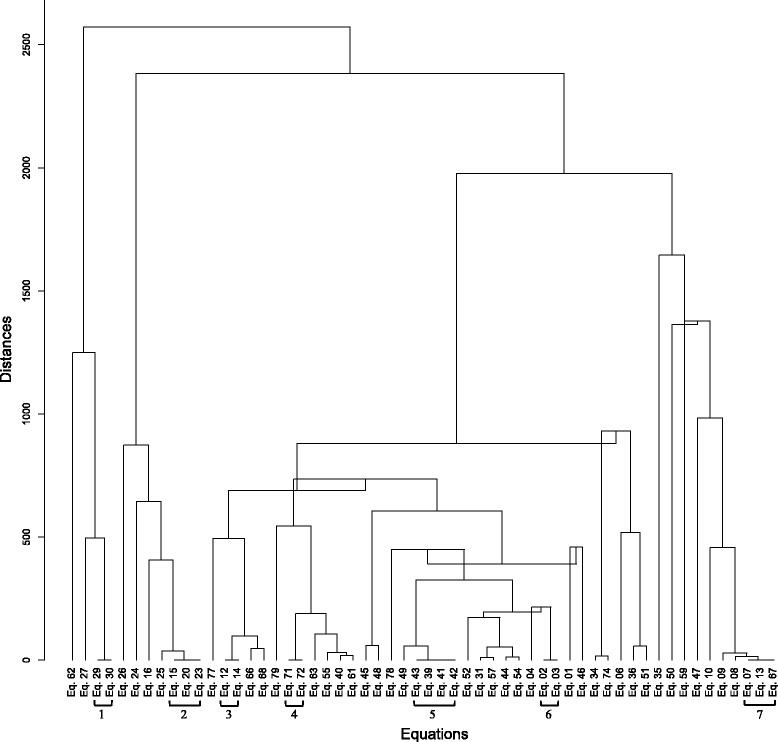
Clustering of 56 binary similarity and dissimilarity measures in the context of Jamu data after removing algebraically redundant equations and equations that produce invalid coefficients. The distances between equations belonging to the same clusters are zero or nearly zero, and we select only one equation from each such cluster for the ROC analysis of the next step

The clustering of 56 equations in the context of Jamu data is shown in Fig. 2. The distances among equations belonging to individual clusters indicated as 1 to 7 in Fig. 2 are equal or nearly equal to 0. In other words, those equations have similar characteristics when generating binary similarity/dissimilarity coefficients for Jamu data. By using the clustering result, we reduced 11 equations denoted by ‘***’ in Table 1 because they were related to other equations in the same cluster e.g. we eliminated S_Baroni-Urbani_&_Buser-2_ (Eq. 72) because it is similar to S_Baroni-Urbani_&_Buser-1_ (Eq. 71). A careful observation of equations belonging to the same cluster in the group IDs 1 to 7 in Fig. 2 implies that one equation can be transformed to another just by adding or multiplying by constants (Table 3). For example, we can represent S_Baroni-Urbani_&_Buser-2_ as [(2 x S_Baroni-Urbani_&_Buser-1_) – 1]. The excluded equations based on the clustering process are as follows: S_Dice-1/Czekanowski_ (Eq. 3), S_Innerproduct_ (Eq. 13), S_Russell_&_Rao_ (Eq. 14), D_Mean-Manhattan_ (Eq. 20), D_Vari_(Eq. 23), D_Chord_ (Eq. 30), S_Kulczynski-2_ (Eq. 41), S_Driver_&_Kroeber_ (Eq. 42), S_Johnson_ (Eq. 43), S_Hamann_ (Eq. 67), and S_Baroni-Urbani_&_Buser-2_ (Eq. 72). In case of Kampo dataset, the clustering results also identified the same equations belong to the same cluster with zero or nearly to zero distance. Therefore, both datasets eliminated the same equations, indicated by ‘***” in Table 1, and also obtained the same number of selected equations (45 binary similarity and dissimilarity measures) for further analysis. Hence, among the 79 binary similarity dissimilarity measures used over the last century, there are only 45 unique equations that produce different coefficients by capturing different information. Additionally, these binary measures satisfy the symmetry property [71], i.e. in case of such equations *d*(*x*, *y*) = *d*(*y*, *x*) or *S*(*x*, *y*) = *S*(*y*, *x*).

**Table 3:** Transformation of an equation into another by adding or multiplying by constants (Group IDs correspond to clusters in Fig. 2)

Group IDs	Eliminated Equations	Selected Equations^a^
1	${D}_{Chord}=\sqrt{2\left(1-\frac{a}{\sqrt{\left(a+b\right)\left(a+c\right)}}\right)}$ (Eq 30)	$=\frac{1}{\sqrt{2}}2\sqrt{\left(1-\frac{a}{\sqrt{\left(a+b\right)\left(a+c\right)}}\right)}=\frac{1}{\sqrt{2}}{D}_{Hellinger}$ (Eq.29)
2	${D}_{Mean- Manhattan}=\frac{b+c}{a+b+c+d}$ (Eq.20)	$=\frac{1}{M}\left(b+c\right)=\frac{1}{M}{D}_{Hamming}$ (Eq.15)
	${D}_{Vari}=\frac{b+c}{4\left(a+b+c+d\right)}$ (Eq.23)	$=\frac{1}{4M}\left(b+c\right)=\frac{1}{4M}{D}_{Hamming}$ (Eq.15)
3	${S}_{Russell\&Rao}=\frac{a}{a+b+c+d}$ (Eq.14)	$=\frac{1}{M}a=\frac{1}{M}{S}_{Intersection}$ (Eq.12)
4	${S}_{Baroni- Urbani\& Buser-2}=\frac{\sqrt{ad}+a-\left(b+c\right)}{\sqrt{ad}+a+b+c}$ (Eq.72)	$=2\frac{\sqrt{ad}+a}{\sqrt{ad}+a+b+c}-1=\left[2 \times {S}_{Baroni- Urbani\& Buser-1}\right]$ ^.^(Eq.71)
5	${S}_{Kulczynski-2}=\frac{\frac{a}{2}\left(2a+b+c\right)}{\left(a+b\right)\left(a+c\right)}$ (Eq.41)	$=\frac{1}{2}\left(\frac{a}{a+b}+\frac{a}{a+c}\right)=\frac{1}{2}{S}_{Johnson}$ (Eq.43)
	${S}_{Driver\& Kroeber}=\frac{a}{2}\left(\frac{1}{a+b}+\frac{1}{a+c}\right)$ (Eq.42)	$=\frac{1}{2}\left(\frac{a}{a+b}+\frac{a}{a+c}\right)=\frac{1}{2}{S}_{Johnson}$ (Eq.43)
	${S}_{Johnson}=\frac{a}{a+b}+\frac{a}{a+c}$ (Eq.43)	$=1+\left(\frac{a^2-bc}{\left(a+b\right)\left(a+c\right)}\right)=1+{S}_{McConnaughey}$ (Eq.39)
6	${S}_{Dice-1/ Czekanowski}=\frac{2a}{2a+b+c}$ (Eq.3)	$=2\frac{a}{2a+b+c}=2 \times {S}_{Dice-2}$ (Eq.2)
7	*S*_*Innerproduct*_ = *a* + *d* (Eq.13)	$=M\frac{a+d}{a+b+c+d}=M \times {S}_{Sokal\& Michener}$ (Eq.7)
	${S}_{Hamann}=\frac{\left(a+d\right)-\left(b+c\right)}{a+b+c+d}$ (Eq.67)	$=2\left(\frac{a+d}{a+b+c+d}\right)-1=\left[2 \times {S}_{Sokal\& Michener}\right]-1$ (Eq.7)

^a^ *M* is a constant (*a* + *b* + *c* + *d*)

**Fig. 3:**
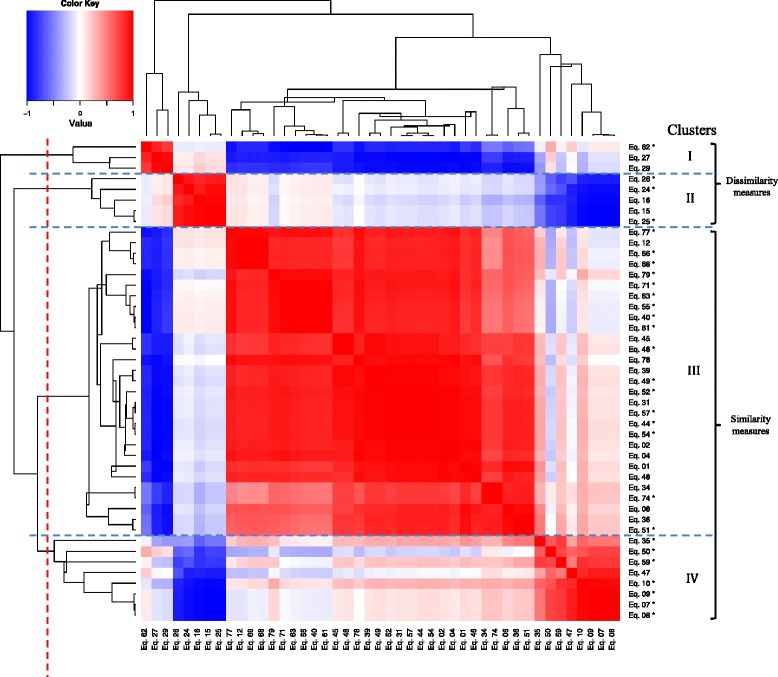
The heatmap and dendrogram of remaining binary similarity and dissimilarity measures using Jamu data. The asterisk symbol (*) indicates the negative match quantity *d* is used in the equation

We applied hierarchical clustering again to these 45 equations to give a better understanding of relationships between selected equations. In general, Jamu and Kampo data generated more or less the same heatmap. The resulted dendrogram together with the heatmap of Jamu data are shown in Fig. 3. We can roughly identify four main clusters (I, II, III, and IV). The hierarchical clustering clearly separated the equations on the basis whether they measure similarity or dissimilarity. Although both similarity/dissimilarity measures may produce the same coefficient range, they work in the opposite way. The higher the similarity between two herbal medicine formulas, the higher the similarity coefficients. On the other hand, the higher the similarity between two herbal medicine formulas the lower the dissimilarity coefficients. Therefore, the agglomerative clustering with centroid linkage performs well in the process to separate similarity and dissimilarity equations. All the equations belonging to clusters I and II are for measuring dissimilarity whereas the equations belonging to clusters III and IV are for measuring similarity. Conversely, the equations that include negative match quantity *d* spread throughout all the clusters. This result indicates that the equations cannot be grouped based on the existence of negative match quantity *d*.

### ROC analysis of selected equations

The ROC curves were created for each binary similarity/dissimilarity equation to compare their performance. Initially, we normalized the similarity and dissimilarity coefficients, such that their minimum becomes 0 and maximum becomes 1, before using them to create the ROC curves. In the case of equations that measure dissimilarity, we transformed a normalized dissimilarity coefficient *D* to a similarity coefficient *S* for the sake of comparison by using the following equation *S* = 1 − *D* ^2^ [40, 41].

In the context of Jamu data, we started the ROC analysis of selected equations by classifying the Jamu pairs into match and mismatch classes based on their efficacies. A Jamu pair belongs to the match class if the efficacy of both the Jamu formulas of a pair is the same. On the other hand, a Jamu pair belongs to the mismatch class if the efficacies of the formulas of a pair are different. The number of Jamu pairs in the match and mismatch classes are 646,728 and 4,253,287 respectively. Obviously, the number of Jamu pairs in the mismatch class is much larger than that in the match class. This imbalance is a challenge in assessment of the capability of equations to separate Jamu pairs into match and mismatch classes. In order to handle this condition, we created 20 mismatch classes each equal to the size of the match class by random sampling of the mismatch class Jamu pairs according to bootstrap method [67]. Every equation was then iteratively evaluated by using those datasets as mismatch class data.

Our objective is to assess the capability of the equations to separate the Jamu pairs into match and mismatch efficacy classes based on their similarity coefficients using ROC analysis. In order to create an ROC curve corresponding to an equation, we need the distributions of match class and mismatch class Jamu pairs with respect to their similarity values calculated by the equation. We divided the range of the similarity coefficient into 100 equal intervals, and the lower limit of each interval was considered as a threshold. Corresponding to every threshold, *TP* and *FN* were determined from the distribution of match class and *FP* and *TN* were determined from the distribution of mismatch class. In our case, *TP* and *FP* are the numbers of Jamu pairs with the similarity value larger than or equal to threshold, and *FN* and *TN* are the numbers of Jamu pairs with the similarity value smaller than threshold. *FPR* and *TPR* were then calculated for every threshold using Eq. 82. We produced the ROC curve by plotting the resulting *FPR* on the *x*-axis and *TPR* on the *y*-axis. In perfect or ideal classification, the ROC curve follows the vertical line from (0,0) to (0,1) and then horizontal line up to (1,1). In the case of random data, the ROC curve follows the diagonal line from (0,0) to (1,1). In the case of real data, the ROC curve usually follows an above diagonal line. The (0,1) is the optimum classification point where *FPR* is zero and *TPR* is one and hence the (0,1) point will be referred to as ‘optimum point’. The performance of a classifier was assessed either by measuring the minimum distance from the optimum point to the curve or by measuring the AUC. In the case of the minimum distance, the lower is the value of the minimum distance the better is the performance of the classifier. In the case of the AUC, the bigger is the AUC value, the better is the performance of the classifier.

**Fig. 4:**
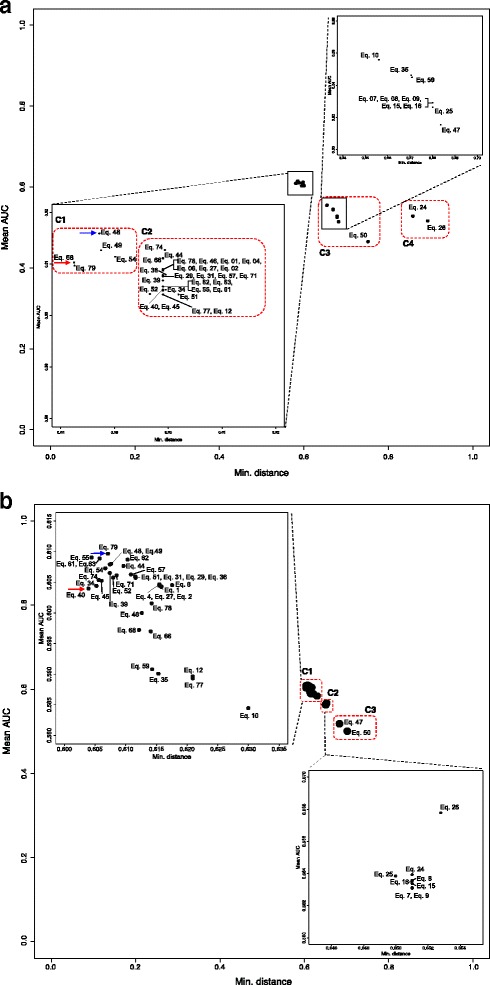
Scatter plot of the minimum distance vs. the mean of area under the ROC curves generated using (**a**) Jamu and (**b**) Kampo data. Red arrow indicates the shortest Euclidean distance between the theoretical optimum point and (*FPR*, *TPR*) points. Blue arrow indicates the highest AUC value

**Fig. 5:**
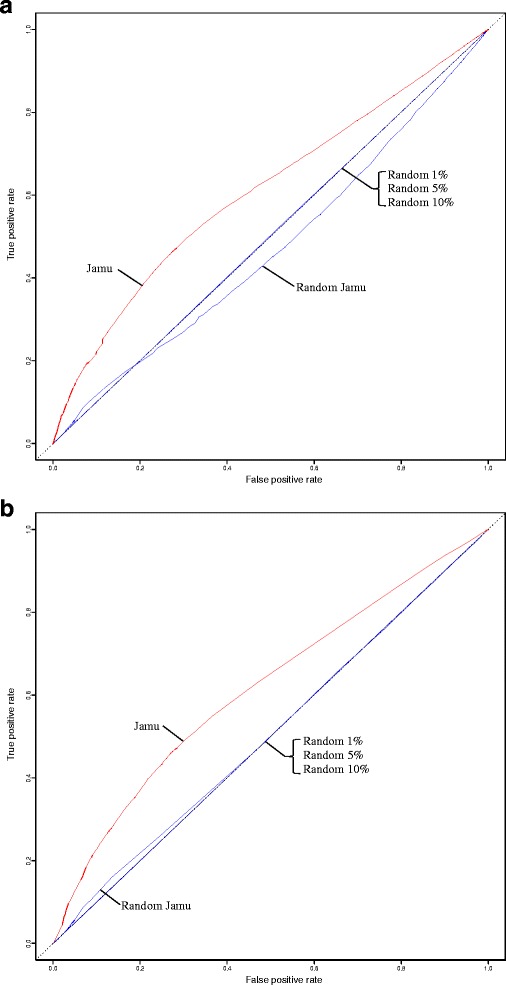
The ROC curves of Michael and Forbes-2 similarities for Jamu and random datasets. **a** Michael similarity (Eq. 68). **b** Forbes-2 similarity (Eq. 48)

In order to assess the effectiveness of an equation using the minimum distance, the ROC curve was generated by using all of the Jamu pairs from match and mismatch efficacies. The Euclidean distance metric was used to measure the distance from the (0, 1) point to the (*FPR*, *TPR*) points for all 45 selected equations. In addition, we created 20 ROC curves for each equation considering in each case the match class Jamu pairs and one of the 20 different mismatch class samples. Thus, we obtained 20 AUCs of the ROC curve for each equation and averaged those values to determine the overall AUCs corresponding to an equation. The ROCR package [72] was used to calculate the AUC values. Table 4 shows the results of ROC analysis and also Kappa scores for Jamu data. The scatter plot of minimum distances and mean of AUCs corresponding to 45 equations for both datasets is shown in Fig. 4. Based on the scatter plot generated using Jamu data in Fig. 4a, the 45 equations are empirically divided into 4 groups (C1, C2, C3, and C4). The well-performing equations corresponding to both approaches were obtained in C1, which consists of Eqs. 48, 49, 54, 68, and 79. The Michael similarity (Eq. 68) produces the lowest minimum distance, and the highest AUC is obtained by the Forbes-2 similarity (Eq. 48). The ROC curves generated using Michael and Forbes-2 similarities for all datasets are shown in Fig. 5. As expected, the ROC curves corresponding to all random datasets follow the diagonal line and that corresponding to Jamu data follows the above diagonal line. Most equations with the highest AUC values are similarity-measuring equations and these equations belong to cluster III in Fig. 3. Out of these equations, the Lance & Williams distance (Eq. 27) produces the highest AUC value among dissimilarity-measuring equations.

**Table 4:** The ROC analysis and Cohen’s Kappa score of Jamu data. A value inside the bracket in the minimum distance and mean Kappa columns represents the ranking of an equation if we order based on respective columns. Standard deviations from both metrics are relatively similar and small, those are 2-4×10^-4^ for mean AUCs and 0-6×10^-4^ for mean of Kappa scores

No	Equations	S/D	Incl. d*	ROC analysis		Cohen’s Kappa
				Mean AUCs	Min. distance	Mean Kappa
1	Eq. 48	S	Y	0.616	0.587 (3)	0.088 (13)
2	Eq. 74	S	Y	0.613	0.599 (29)	0.024 (28)
3	Eq. 49	S	Y	0.613	0.588 (4)	0.076 (15)
4	Eq. 54	S	Y	0.611	0.590 (5)	0.074 (19)
5	Eq. 44	S	Y	0.611	0.599 (19)	0.073 (21)
6	Eq. 66	S	Y	0.611	0.599 (26)	0.023 (31)
7	Eq. 68	S	Y	0.610	0.583 (1)	0.024 (29)
8	Eq. 79	S	Y	0.610	0.583 (2)	0.090 (11)
9	Eq. 78	S		0.609	0.599 (28)	0.092 (8)
10	Eq. 46	S		0.609	0.599 (20)	0.065 (23)
11	Eq. 01	S		0.609	0.599 (10)	0.052 (24)
12	Eq. 04	S		0.609	0.599 (11)	0.089 (12)
13	Eq. 06	S		0.609	0.599 (12)	0.036 (27)
14	Eq. 27	D		0.609	0.599 (14)	0.109 (7)
15	Eq. 02	S		0.609	0.599 (8)	0.074 (20)
16	Eq. 36	S		0.608	0.600 (31)	0.040 (25)
17	Eq. 29	D		0.608	0.599 (15)	0.076 (16)
18	Eq. 31	S		0.608	0.599 (16)	0.076 (17)
19	Eq. 57	S	Y	0.608	0.599 (22)	0.076 (18)
20	Eq. 71	S	Y	0.608	0.599 (9)	0.152 (6)
21	Eq. 39	S		0.607	0.599 (17)	0.078 (14)
22	Eq. 62	D	Y	0.606	0.599 (24)	0.185 (1)
23	Eq. 63	S	Y	0.606	0.599 (25)	0.167 (5)
24	Eq. 55	S	Y	0.606	0.599 (21)	0.180 (3)
25	Eq. 61	S	Y	0.606	0.599 (23)	0.183 (2)
26	Eq. 40	S	Y	0.605	0.599 (18)	0.180 (4)
27	Eq. 34	S		0.605	0.600 (30)	0.024 (30)
28	Eq. 45	S		0.605	0.599 (7)	0.091 (10)
29	Eq. 52	S	Y	0.604	0.597 (6)	0.092 (9)
30	Eq. 77	S	Y	0.604	0.599 (27)	0.067 (22)
31	Eq. 51	S	Y	0.604	0.602 (32)	0.039 (26)
32	Eq. 12	S		0.604	0.599 (13)	0.022 (32)
33	Eq. 10	S	Y	0.556	0.656 (33)	0.014 (34)
34	Eq. 35	S	Y	0.546	0.671 (34)	0.018 (33)
35	Eq. 59	S	Y	0.545	0.671 (35)	0.013 (35)
36	Eq. 24	D	Y	0.529	0.860 (44)	0.000 (43)
37	Eq. 15	D		0.529	0.680 (39)	0.004 (42)
38	Eq. 08	S	Y	0.529	0.680 (37)	0.010 (39)
39	Eq. 09	S	Y	0.529	0.680 (38)	0.010 (36)
40	Eq. 16	D		0.529	0.680 (40)	0.010 (38)
41	Eq. 07	S	Y	0.529	0.680 (36)	0.010 (37)
42	Eq. 25	D	Y	0.526	0.680 (41)	0.004 (41)
43	Eq. 26	D	Y	0.517	0.895 (45)	0.000 (44)
44	Eq. 47	S		0.515	0.684 (42)	0.005 (40)
45	Eq. 50	S	Y	0.466	0.754 (43)	-0.008 (45)

The column "Incl. *d*" means the availability of negative match quantity d in the equation (Yes/No)

We repeated our experiments also for Kampo data following the same procedures. The results of ROC analysis and also Cohen’s Kappa using Kampo data are shown in Table 5. In addition, the plot between minimum distances and mean AUCs of Kampo data is shown in Fig. 4b. The remaining equations are clustered into 3 groups (C1, C2 and C3). The most suitable binary equations for classifying Kampo data were found in the cluster C1, with Tarwid Similarity (Eq. 40) and Variant of Correlation similarity (Eq. 79) producing the lowest minimum distance and the highest mean AUCs, respectively, which are different from the top ranking equations in case of Jamu data. Only 5 of top-10 well-performing equations corresponding to Jamu data matches with those corresponding to Kampo data with different order. These results indicate different dataset produce different ranking of equations and there is no superior equation that can perform well for all datasets [73]. Each binary similarity and dissimilarity equation has its own characteristics and fits for a specific problem. Therefore, our proposed method can be used to choose the appropriate equations wisely, depending on the characteristics of the data to analyze.

**Table 5:** The ROC analysis and Cohen’s Kappa score of Kampo data. A value inside the bracket in the minimum distance and mean Kappa columns represents the ranking of an equation if we order based on respective columns

No	Equations	S/D	Incl. d*	ROC analysis			Cohen’s Kappa	
				Mean AUCs	SD mean AUCs	Min. distance	Mean Kappa	SD mean Kappa
1	Eq. 79	S	Y	0.610	0.001	0.607 (9)	0.069 (14)	0.001
2	Eq. 55	S	Y	0.609	0.001	0.604 (2)	0.106 (1)	0.001
3	Eq. 61	S	Y	0.609	0.001	0.606 (5)	0.106 (2)	0.001
4	Eq. 63	S	Y	0.609	0.001	0.606 (6)	0.099 (5)	0.001
5	Eq. 62	D	Y	0.609	0.001	0.610 (16)	0.101 (4)	0.001
6	Eq. 48	S	Y	0.608	0.001	0.608 (12)	0.084 (9)	0.001
7	Eq. 49	S	Y	0.608	0.001	0.607 (11)	0.069 (15)	0.001
8	Eq. 44	S	Y	0.608	0.001	0.610 (15)	0.065 (21)	0.001
9	Eq. 54	S	Y	0.607	0.001	0.607 (8)	0.066 (20)	0.001
10	Eq. 39	S		0.607	0.002	0.607 (10)	0.070 (13)	0.001
11	Eq. 57	S	Y	0.606	0.001	0.611 (17)	0.067 (18)	0.000
12	Eq. 71	S	Y	0.606	0.001	0.608 (14)	0.092 (6)	0.001
13	Eq. 51	S	Y	0.606	0.001	0.612 (18)	0.040 (27)	0.001
14	Eq. 31	S		0.606	0.001	0.612 (20)	0.068 (17)	0.001
15	Eq. 29	D		0.606	0.001	0.612 (19)	0.068 (16)	0.001
16	Eq. 52	S	Y	0.606	0.001	0.608 (13)	0.078 (10)	0.001
17	Eq. 36	S		0.606	0.001	0.612 (21)	0.042 (26)	0.001
18	Eq. 74	S	Y	0.605	0.002	0.606 (4)	0.037 (29)	0.001
19	Eq. 45	S		0.605	0.001	0.606 (7)	0.086 (8)	0.001
20	Eq. 04	S		0.605	0.001	0.615 (29)	0.075 (12)	0.001
21	Eq. 27	D		0.605	0.001	0.615 (30)	0.091 (7)	0.001
22	Eq. 06	S		0.605	0.001	0.618 (32)	0.032 (40)	0.001
23	Eq. 02	S		0.604	0.001	0.615 (28)	0.065 (22)	0.001
24	Eq. 34	S		0.604	0.001	0.605 (3)	0.035 (36)	0.001
25	Eq. 01	S		0.604	0.001	0.616 (31)	0.047 (24)	0.001
26	Eq. 40	S	Y	0.604	0.001	0.604 (1)	0.102 (3)	0.002
27	Eq. 78	S		0.602	0.001	0.614 (25)	0.075 (11)	0.001
28	Eq. 46	S		0.600	0.001	0.613 (23)	0.055 (23)	0.001
29	Eq. 68	S	Y	0.597	0.001	0.612 (22)	0.036 (32)	0.001
30	Eq. 66	S	Y	0.597	0.001	0.614 (24)	0.035 (37)	0.001
31	Eq. 59	S	Y	0.591	0.001	0.614 (26)	0.043 (25)	0.001
32	Eq. 35	S	Y	0.590	0.001	0.615 (27)	0.036 (35)	0.001
33	Eq. 12	S		0.590	0.001	0.621 (33)	0.034 (38)	0.000
34	Eq. 77	S	Y	0.589	0.001	0.621 (34)	0.066 (19)	0.000
35	Eq. 10	S	Y	0.584	0.001	0.630 (35)	0.036 (31)	0.001
36	Eq. 26	D	Y	0.568	0.001	0.653 (43)	0.015 (43)	0.001
37	Eq. 24	D	Y	0.564	0.001	0.651 (42)	0.017 (42)	0.001
38	Eq. 25	D	Y	0.564	0.001	0.650 (36)	0.032 (41)	0.001
39	Eq. 08	S	Y	0.564	0.001	0.651 (38)	0.036 (33)	0.001
40	Eq. 16	D		0.564	0.001	0.651 (41)	0.037 (30)	0.001
41	Eq. 15	D		0.563	0.001	0.651 (40)	0.032 (39)	0.001
42	Eq. 07	S	Y	0.563	0.001	0.651 (37)	0.036 (34)	0.001
43	Eq. 09	S	Y	0.563	0.001	0.651 (39)	0.037 (28)	0.001
44	Eq. 47	S		0.518	0.001	0.683 (44)	0.010 (44)	0.001
45	Eq. 50	S	Y	0.501	0.001	0.702 (45)	-0.004 (45)	0.000

The column "Incl. *d*" means the availability of negative match quantity d in the equation (Yes/No)

In case of Jamu and Kampo pairs, the negative match quantity *d* is much higher compared to the positive match *a* and the absence mismatches *b* and *c*. One of our objectives is to understand the effect of *d* in calculating similarity/dissimilarity coefficients between herbal medicines. Among the equations that do not include *d*, the Simpson similarity (Eq. 45) and the Forbes-1 similarity (Eq. 34) produce the lowest minimum distance in Jamu and Kampo data, respectively. Furthermore, the Derived Jaccard similarity (Eq. 78) and the McConnaughey (Eq. 39) produce the highest AUC in Jamu data and Kampo data. Out of 79 equations in Table 1, 46 equations use *d* in their expressions. Interestingly, the equations that include *d* perform better in measuring similarity/dissimilarity in both datasets. The best performing equations corresponding to minimum distance and mean AUCs for Jamu data are Eqs. 68 and 48, which include negative match quantity *d*. Likewise, the best equations in the Kampo data (Eqs. 79 and 40) also include negative match quantity *d*. Then, the top-5 well performing equations corresponding to both datasets include *d*. If we also consider another metric to rank the classifier performance, i.e. Cohen’s Kappa, we find a consistent result. That is top-5 equations with the largest Kappa score also include *d* (Table 4 and 5). It implies the similarity between Jamu pairs and Kampo pairs are influenced by the negative matches. This result supports the findings of Zhang et al. [20] that all possible matches, *S*_*ij*_ where *i, j* ϵ{0,1}, should be considered for better classification results. Moreover, the performance measurement of binary similarity/dissimilarity equations using the AUC of ROC curve is more preferable to the minimum distance because this approach considers all (*FPR*, *TPR*) points, not only a single point with minimum distance to the optimum point.

**Fig. 6:**
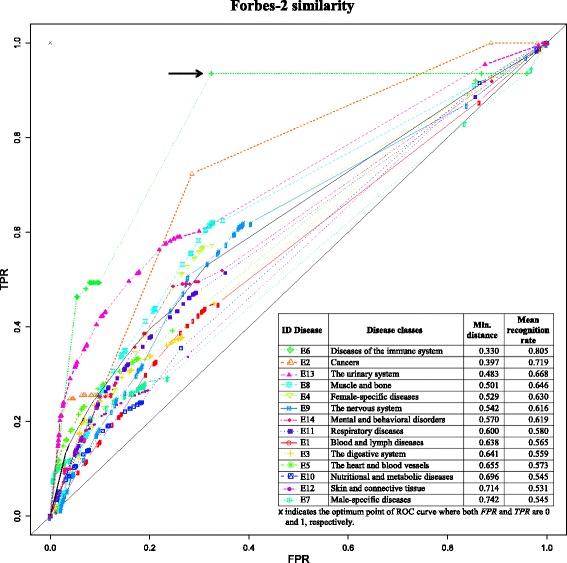
The ROC curves for every disease class in Jamu data using Forbes-2 similarity coefficient. The average of recognition rate was calculated as $\frac{1}{2}\left[\frac{TP}{TP+FN}+\frac{TN}{TN+FP}\right]$ by using the *TP*, *FN*, *FP*, and *TN* values from (*FPR*, *TPR*) point with the shortest distance to the optimum point (0,1)

For further insight into the matter, we examined the performance of the equations for every disease class in Jamu data separately using the same approach. We created match and mismatch datasets for every disease class using all Jamu pairs. The match class consists of Jamu pairs with the same efficacy class and the mismatch class consists of Jamu pairs with different efficacy class but one of the Jamu formulas in that pair has the same efficacy class as the match class. To measure the AUC of ROC curve, we created 20 mismatch classes each equal to the size of the match class by using the bootstrap method. Thus, we obtained 20 AUCs of the ROC curves for each disease class and each equation, and we averaged those 20 values to determine the overall AUCs corresponding to a disease class and an equation (Additional file 1: Table S1). Figure 6 shows the ROC curves for every disease class using Forbes-2 similarity coefficients. The immune system disease class (E6) produces the highest AUC score and the highest average of AUCs (for all 45 equations). Moreover, the best classification is obtained in case of immune system class indicated by an arrow in Fig. 6, with the average of recognition rate of 0.805. The relatively high recognition rate of E6 class corresponds to our knowledge that the disease of immune system class is a very specific disease and utilization of the crude drug is restricted compared to other disease classes. The minimum distance of an ROC curve from the optimum point (expressed by Eq. 83) indicates the difficulty of classification i.e. the higher the minimum distance the more difficult it is to achieve a successful classification. Therefore, when the minimum distance is close to zero, it implies that good classification of the data is possible. In case of classification of Jamu formulas concerning individual diseases, relatively lower minimum distance was obtained for specific type of disease classes such as diseases related to E6 and the urinary systems (E13), which indicates that very specific types of medicinal plants are used to make such Jamu formulas. On the other hand, the disease classes such as those related to digestive systems (E3) and nutritional and metabolic diseases (E10) are caused by diverse factors and therefore the corresponding Jamu formulas are made using diverse types of plants resulting in relatively higher minimum distance for these disease classes (Fig. 6).

## Conclusions

Different binary similarity and dissimilarity measures yield different similarity/dissimilarity coefficients, which in turn causes differences in downstream analysis e.g. clustering. Hence, determining appropriate binary similarity and dissimilarity coefficients is an essential aspect of big data analysis in versatile areas of scientific research including chemometrics and bioinformatics. In this study, we presented an organized way to select a suitable equation for studying relationship between herbal medicine formulas in Indonesian Jamu and Japanese Kampo. We started our study by collecting 79 binary similarity and dissimilarity equations from literature. In the early stages, we reduced algebraically redundant equations and equations that produce invalid values or relatively similar coefficients when applied to our datasets. In addition, we eliminated some equations based on agglomerative hierarchical clustering because they were very closely related to other equations in the same cluster. Finally, we selected 45 unique equations that produced different coefficients for our analysis. The ROC curve analysis was then performed to assess the capabilities of these equations to separate herbal medicine pairs having the same and different efficacies. The experimental results show that the binary similarity and dissimilarity measures that include the negative match quantity *d* in their expressions have a better capability to separate herbal medicine pairs than those equations that exclude *d*. Moreover, we obtained different ranking of binary equations for different datasets, i.e. Jamu and Kampo data. Thus, this result indicates the selection of binary similarity and dissimilarity measures is data dependent and we should choose the binary similarity and dissimilarity measures wisely depending on the data to be processed. In case of Jamu data, the biggest AUC value is obtained by the Forbes-2 similarity. Conversely, the Variant of Correlation similarity is recommended for classifying Kampo pairs into match and mismatch classes. The procedure followed in this work can also be used to find suitable binary similarity and dissimilarity measures under similar situations in other applications.

## Additional file

Additional file 1: Table S1. The mean of AUCs between equations and disease classes in Jamu data. (XLSX 50 kb)

## Abbreviations

AUC: The Area Under the ROC Curve
D: Dissimilarity
FN: False Negative
FP: False Positive
FPR: False Positive Rate
NA-DFC: The National Agency of Drug and Food Control
NCBI: The National Center for Biotechnology Information
OTU: The Operational Taxonomic Unit
PR: Precision-Recall
ROC: The Receiver Operating Characteristic
S: Similarity
TCM: Traditional Chinese Medicine
TN: True Negative
TP: True Positive
TPR: True Positive Rate
